# Temporal Profiling of Gene Networks Associated with the Late Phase of Long-Term Potentiation *In Vivo*

**DOI:** 10.1371/journal.pone.0040538

**Published:** 2012-07-10

**Authors:** Margaret M. Ryan, Brigid Ryan, Madeleine Kyrke-Smith, Barbara Logan, Warren P. Tate, Wickliffe C. Abraham, Joanna M. Williams

**Affiliations:** 1 Brain Health Research Centre, University of Otago, Dunedin, New Zealand; 2 Department of Anatomy, Otago School of Medical Sciences, Dunedin, New Zealand; 3 Department of Biochemistry, Otago School of Medical Sciences, Dunedin, New Zealand; 4 Department of Psychology, University of Otago, Dunedin, New Zealand; The George Washington University, United States of America

## Abstract

Long-term potentiation (LTP) is widely accepted as a cellular mechanism underlying memory processes. It is well established that LTP persistence is strongly dependent on activation of constitutive and inducible transcription factors, but there is limited information regarding the downstream gene networks and controlling elements that coalesce to stabilise LTP. To identify these gene networks, we used Affymetrix RAT230.2 microarrays to detect genes regulated 5 h and 24 h (n = 5) after LTP induction at perforant path synapses in the dentate gyrus of awake adult rats. The functional relationships of the differentially expressed genes were examined using DAVID and Ingenuity Pathway Analysis, and compared with our previous data derived 20 min post-LTP induction *in vivo*. This analysis showed that LTP-related genes are predominantly upregulated at 5 h but that there is pronounced downregulation of gene expression at 24 h after LTP induction. Analysis of the structure of the networks and canonical pathways predicted a regulation of calcium dynamics via G-protein coupled receptors, dendritogenesis and neurogenesis at the 5 h time-point. By 24 h neurotrophin-NFKB driven pathways of neuronal growth were identified. The temporal shift in gene expression appears to be mediated by regulation of protein synthesis, ubiquitination and time-dependent regulation of specific microRNA and histone deacetylase expression. Together this programme of genomic responses, marked by both homeostatic and growth pathways, is likely to be critical for the consolidation of LTP *in vivo.*

## Introduction

Long-term potentiation (LTP) is widely regarded as a memory storage mechanism as it fulfils key properties of a mnemonic device. For example, LTP is specific to only closely grouped synapses activated following high-frequency stimulation [1], [2]; LTP shows both cooperativity and associativity, such that for LTP induction a threshold intensity is required, as is coincident activity of the pre- and postsynaptic neurons. LTP is also remarkably persistent, when induced at perforant path synapses of awake freely moving animals, LTP has been shown to transition through distinct phases which culminate in a potentiation that can remain stable for months and in one documented case, even for a year [3]. The molecular mechanisms underlying the early events have been thoroughly characterised both *in vitro* and *in vivo*. Protein kinase activation and subsequent trafficking of AMPA-glutamate receptors to the cell surface, together with altered release of neurotransmitter [4], underpin the early phase of LTP. This is followed by trafficking of NMDA-glutamate receptors [5] and metabotropic glutamate receptors [6] that facilitate a change in the calcium dynamics of activated synapses and reflect metaplasticity events [7].

There is considerable evidence that late phases of LTP (LTP2 and LTP3) require new protein synthesis, both rapidly via regulated translation of synaptically located mRNA and over the long-term via new gene transcription [8]. The role that new gene expression plays in LTP has been debated, however, and there is evidence that LTP can persist under certain *in vitro* conditions without protein synthesis [9], [10], [11]. LTP-related gene expression might contribute indirectly to replenish proteins depleted in response to synaptic activity, while LTP itself is maintained by enzymatic storage mechanisms [12]. Nonetheless, evidence primarily from studies of LTP *in vivo* strongly supports the hypothesis that new gene expression is crucial for the persistence of LTP. General inhibitors of transcription and translation or knockdown of specific LTP-regulated genes inhibit the maintenance of LTP *in vivo*, and clusters of genes have been identified as regulated by LTP-inducing stimulation [13], [14], [15]. Of these genes, those that rapidly respond fall into two categories: inducible transcription factors (e.g. members of the EGR and AP1 families) and effector genes (e.g. ARC and HOMER1A), suggesting that new gene expression contributes to LTP maintenance by different modalities. However, the temporal organization of the LTP-related late-gene expression, as driven by the constitutive and inducible transcription factors, has yet to be determined.

Our proposal is that to fully understand the complex molecular mechanisms underlying LTP, it is essential to carry out genome-wide analyses to identify and interpret the gene networks regulated by the biology. Accordingly, we studied the early phase of LTP to identify interrelated networks of genes induced at perforant path synapses in the dentate gyrus *in vivo* [13]. This study and others consolidated the concept that a significant and co-ordinated alteration in gene expression is triggered during LTP [16], [17]. However, few analyses have extended beyond this very early time frame (e.g. [18]). In the current study, we have broadened our analysis to identify gene sets differentially regulated during the late phases of LTP (5 h and 24 h post-LTP induction), elucidating their associated canonical pathways, biological functions and clustering into gene networks. The resulting holistic view of gene expression changes over 24 h has provided insight into the regulation and function of LTP-related gene expression in the hippocampus.

## Analysis

### 1. Regulation of gene expression 20 min, 5 h and 24 h following LTP induction *in vivo*

**Figure 1:**
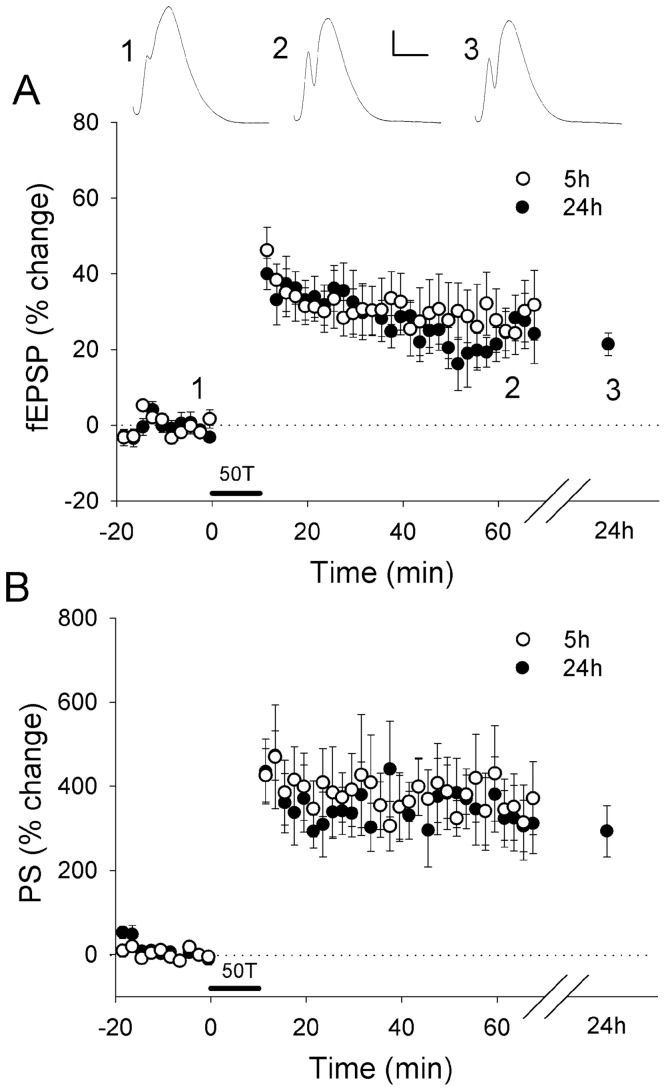
Induction of robust LTP by 50×400 **Hz, 10 impulse trains (HFS) at perforant path dentate granule cell synapses.** Data are mean ± standard error (n = 5) for the fEPSP (A) and population spike (B). Inset waveforms are averages of 10 sweeps (5 min) for one animal, taken during baseline (1), 45–60 min (2) and 24 h (3) after HFS. Calibration bars: 5 ms, 5 mV.

**Figure 2:**
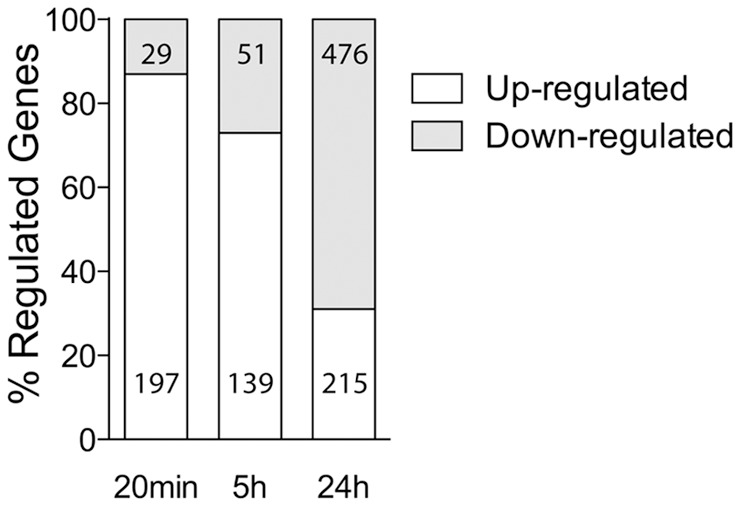
LTP-related gene expression 20 min, 5 **h and 24** **h post-LTP induction.** Alterations in mRNA transcript number identified 20 min, 5 h and 24 h post-LTP, as detected using Affymetrix RAT 230 2.0 microarrays; note increase in proportion of downregulated genes at 24 h (dual selection criteria: fold change ≥1.15 or ≤0.85; p<0.05).

**Figure 3:**
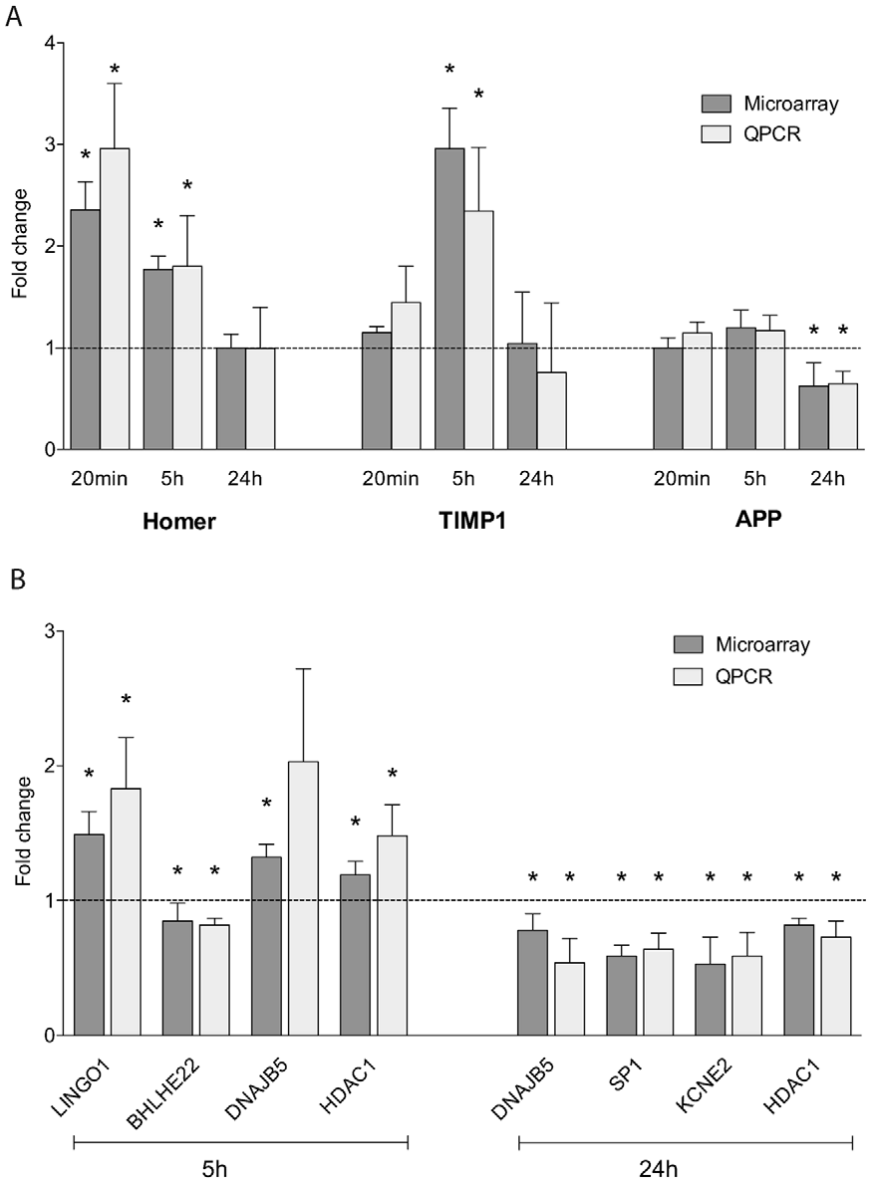
Validation of differentially expressed genes by quantitative PCR (qPCR). Differential expression of selected genes was validated by qPCR. (A) Genes previously reported to be LTP-regulated: HOMER, homer homolog 1; TIMP1, tissue inhibitor of metalloproteinase 1; APP, amyloid precursor protein. (B) Novel LTP-regulated genes: LINGO1, leucine rich repeat and Ig domain containing; SP1 specificity protein 1; BHLHE22, basic helix-loop-helix family, member e22; HDAC1, histone deacetylase 1; KCNE2, potassium voltage-gated channel, Isk-related family, member 2 and DNAJB5, DnaJ (Hsp40) homolog, subfamily B, member 5. Results are expressed as mean fold change ± SEM. *p≤0.05, Student's t-test (n = 5–9). qPCR samples were normalised to the housekeeping gene HPRT using the 2^− ΔΔ^CT method.

To determine whether there are ongoing LTP-related alterations in gene expression and whether overlapping or distinct gene sets are regulated in the later stages of LTP, we established time-specific LTP-related gene (LRG) datasets. Our established LTP induction paradigm is well documented to produce a very persistent form of LTP at perforant path synapses *in vivo* (e.g. [3]). In this study, HFS resulted in a robust potentiation over 24 h of the fEPSP slope (21±3%; mean ± SEM) and the population spike (293±61%) (Figure 1). LRGs were identified by isolation and purification of total RNA from individual control and stimulated hemispheres (5 h and 24 h post-LTP induction; n = 5), followed by gene expression profiling using Affymetrix Rat 230.2 arrays. After normalization across all datasets and using a moderated paired t-test (*Limma*), an inclusive list of differentially expressed LRGs was produced for each time point using dual selection criteria (±1.15 fold change; p<0.05). This analysis identified 190 differentially expressed genes (73% increased and 27% decreased) at 5 h post-LTP induction, and 691 differentially expressed genes at 24 h post-LTP induction (31% increased and 69% decreased) (Figure 2; Table S1, S2). Overall our analysis, when compared with a re-analysis of our 20 min-LRG set [13], suggests that there is a rapid upregulation of gene expression which persists for at least 5 h, but by 24 h the proportion of upregulated genes is attenuated, and a generalised downregulation of gene expression (Figure 2). While little literature exists regarding the specific cohorts of genes regulated in the later phases of LTP [14], [18], [19], we explored our datasets for previously reported LTP-regulated genes. Several known LTP-regulated genes were identified within our new datasets, and a subset was confirmed using qPCR as having temporally specific gene expression (Figure 3A). We confirmed the upregulation of the immediate-early gene HOMER, observed that the metallopeptidase inhibitor TIMP1 is upregulated with a delayed time-course, and showed that the amyloid precursor protein (APP) is downregulated specifically 24 h post-LTP induction. Next, we confirmed the differential expression of several genes, which had not previously been reported to be regulated by LTP (Figure 3B). These included LINGO1, a member of the Nogo receptor signalling complex which regulates axon regeneration, the transcription factors SP1 and BHLHE22, HDAC1, a histone deacetylase, KCNE2, an ancillary protein of voltage-gated potassium channels as well as DNAJB5 (see below).

**Figure 4:**
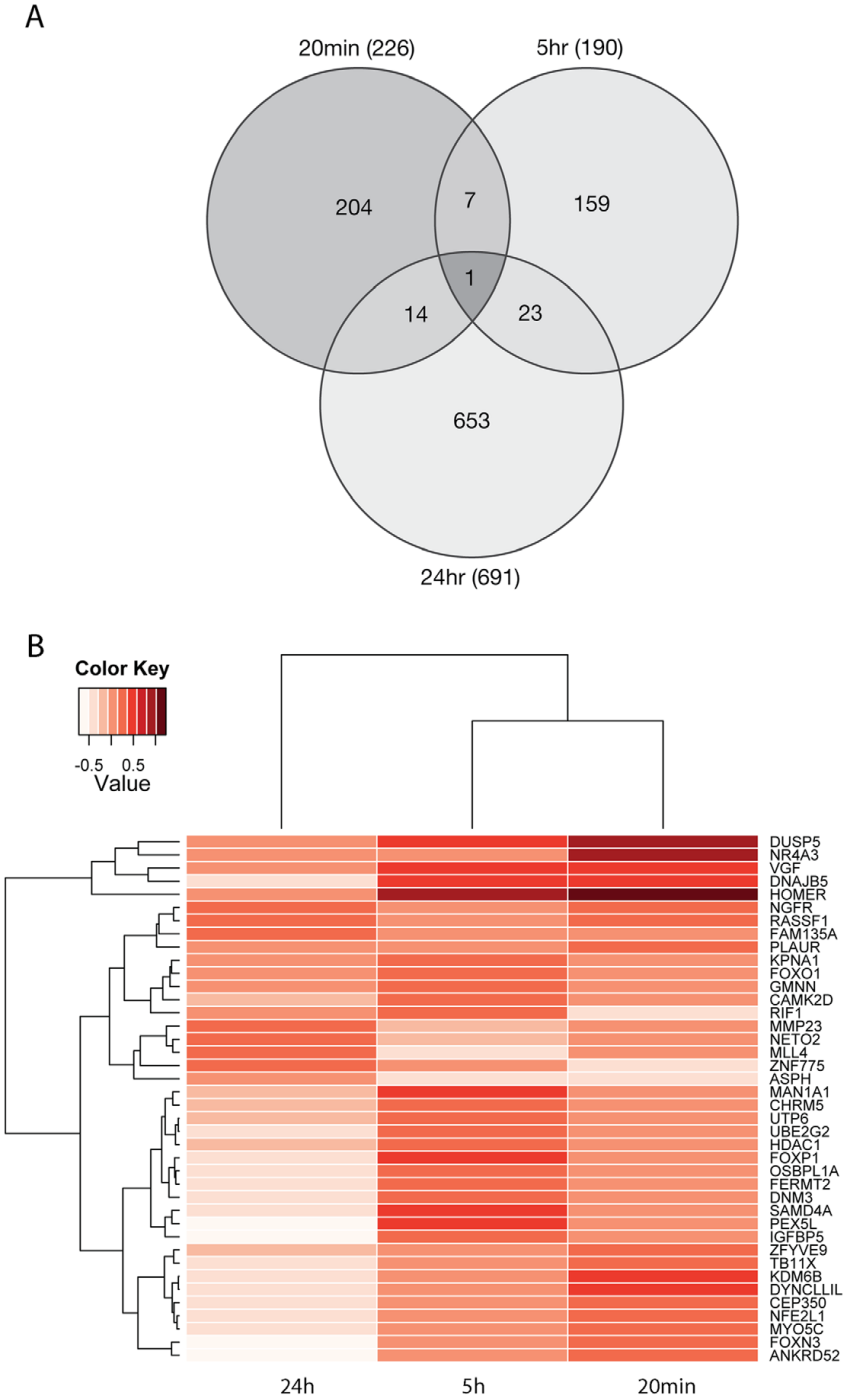
Temporal relationship of LTP-regulated datasets. (A) Venn Diagram showing 3 datasets largely contain temporally specific genes. (B) Heatmap displaying average gene expression values (expressed as log fold change) of overlapping genes across the timepoints.

To determine the overall similarity between our datasets we used Venn diagrams and investigated the intersections formed (Figure 4A; Table S3). This analysis showed that while the 20 min and 5 h datasets contain similar numbers of differentially expressed genes, few are in common and at 24 h there are 3 fold more downregulated genes than at the earlier time points. Analysis of the temporal expression profiles of the genes common between the 3 data sets by heatmap (Figure 4B) demonstrates that there is a close relationship between the 20 min and 5 h data sets; however, the heatmap includes data that have not reached statistical significance and only comprises a small proportion of the differentially expressed genes within each timepoint. Thus we conclude that the three LRG sets represent distinct temporally defined datasets. Remarkably, there is only one gene in common between all 3 data sets, the class II histone deacetylases (HDACs)-interacting protein, DNAJB5 [20]. DNAJB5 is postulated to act as a chaperone involved in nuclear localisation of HDACs, which regulate gene transcription by removal of acetyl groups from ε-N-acetylated histones. This enhances histone-DNA association and prevents access of transcriptional machinery to DNA. Interestingly, also present within the intersection between the 5 h and 24 h gene sets is the class I histone deacetylase HDAC1, GMNN (Geminin), a molecule that has recently been shown to regulate the conformation of chromatin around neural genes [21] and MLL4, a histone methyltransferase which also regulates acetylation [22]. These findings highlight potential epigenetic control of LTP-related gene expression.

### 2. Temporal analysis of LRG sets

To explore the potential contribution that our temporally defined LRG datasets make to the late phases of LTP, the relationships between the contributing genes were analysed using Ingenuity Pathway Analysis (as described [13]) (IPA; Canonical Pathways, Biological Function and Network Analysis), and DAVID software. Our 20 min-LRG data set [13] was reanalysed alongside those from 5 h and 24 h to examine LTP-associated functions across our full temporal range using the most current versions of the software.

#### 2.1 Ingenuity Pathway Analysis: Biological Functions

**Figure 5:**
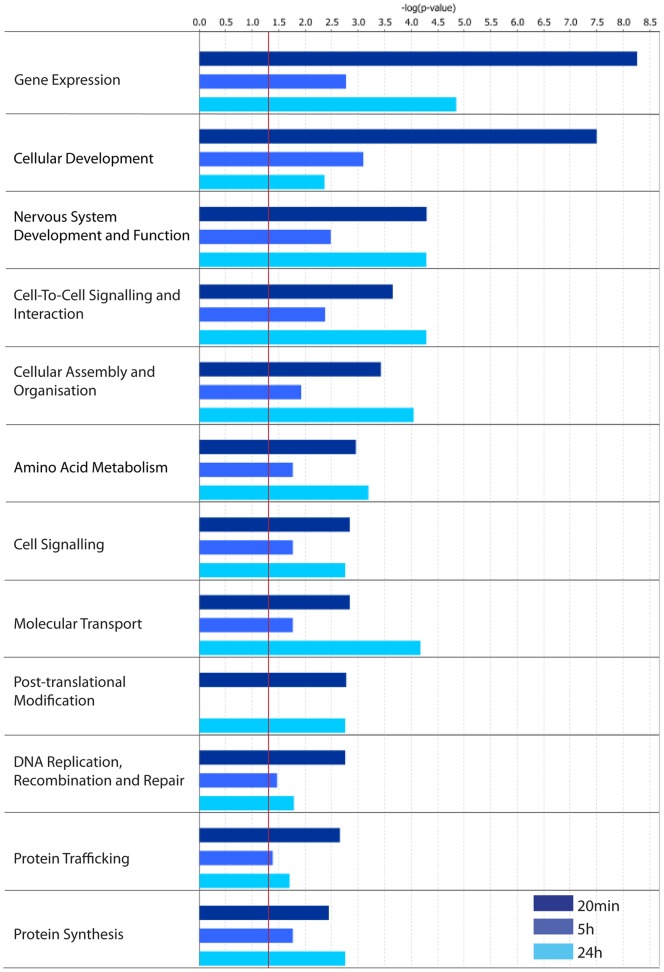
IPA Categorization of LRG sets according to their Biological Functions.

Biological Function analysis (Figure 5) highlighted the contribution that regulated gene expression plays in the maintenance of LTP. The function *Gene expression* was well above the threshold value within all three LRG datasets. Interestingly, *Cell-Cell Signalling & Interaction, Molecular Transport* and *Protein Synthesis* were all more significantly regulated at 24 h. Examination of the genes represented within the latter set (Table S4) indicate a potential downregulation of protein synthesis mediated via regulation of the pre-initiation complex and charging of tRNA with their cognate amino acids (EIF2, EPRS, EIF3) as well as a generalized disruption of the 40S initiation complex via reduction in levels of the scaffolding factor, eIF4G.

#### 2.2 Ingenuity Pathway Analysis: Canonical Pathway Analysis and the Database for Annotation, Visualization and Integrated Discovery

**Figure 6:**
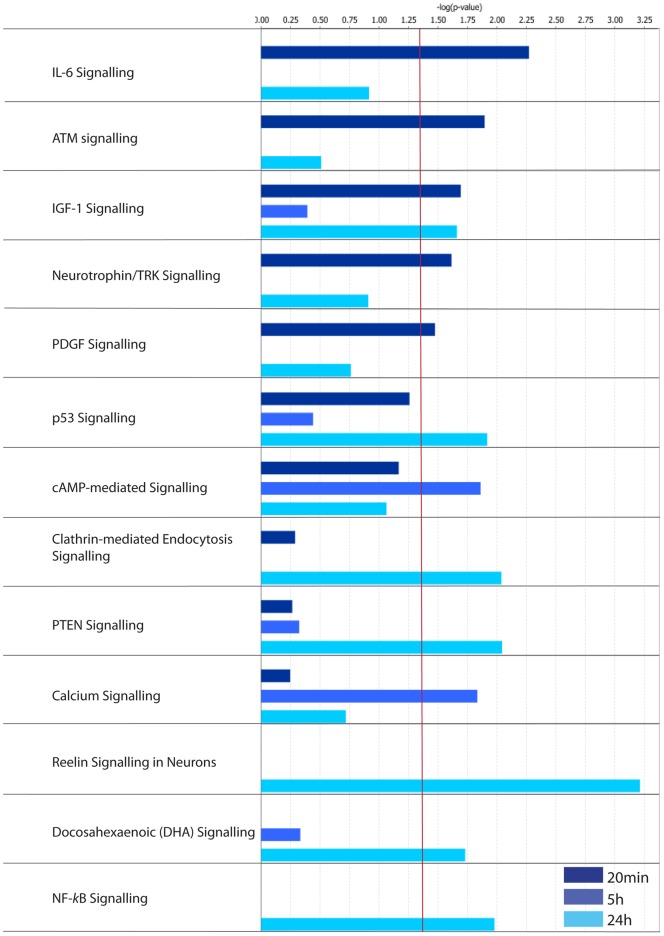
IPA Categorization of LRG sets according to their Canonical Pathways.

The Canonical Pathways identified within the 20 min-LRG set analysis were associated with *Cell growth, Cell death, Development* and *PDGF* signalling (Figure 6). Interestingly, interleukin 6 (IL6) signalling was identified specifically within our 20 min-LRG set. This supports the observation that immune response-related genes are implicated in the LTP stabilization process [16]. Canonical analysis also highlighted an early involvement of *Neurotrophin/TRK* signalling in the LTP stabilization process, as previously described [23], [24].

Canonical pathway analysis of the 5 h-LRG set identified two separate pathways associated with G-protein coupled signalling and calcium dynamics (*cAMP, Ca^2+^ signalling*), with the majority of the genes upregulated (Figure 6; Table S5). In support of these findings, when the 5 h-LRG set was submitted to functional analysis using DAVID software, a *Calcium and Calmodulin* annotation cluster was identified as significantly enriched (enrichment score: 1.42; see Figure S1). Furthermore, DAVID analysis identified clusters of genes with annotations related to *Ion channels* (enrichment score: 1.59; see Figure S2) and *Synapse-related proteins* (enrichment score: 1.37; see Figure S3). DAVID analysis of our 24 h-LRG-set highlighted a significant regulation of *protein kinase* and *phosphorylation*-related genes (enrichment scores 1.98, 2.58 respectively; Figure S4, S5). Closer analysis of the 24 h-LRG-set showed that it contained 39 genes encoding protein kinases, 25 of which were downregulated, as well as 11 phosphatases, 9 of which were downregulated. Thus the DAVID analysis predicts regulation of cAMP and G-protein coupled receptor linked pathways 5 h post-LTP that is followed by downregulation of protein kinases/phosphatases at 24 h. Together these analyses predict that gene expression underpins ongoing modulation of pivotal cell-signalling systems, potentially contributing to a metaplastic shift in calcium dynamics or signalling [25].

Canonical pathway analysis of the 24 h-LRG set identified *NF*K*B* (nuclear factor kappa B), *Reelin*, *PTEN* (phosphatase and tensin homolog), *DHA* (omega-3 fatty acid DHA; docosahexaenoic acid) and *Clathrin-mediated endocytosis* pathways as specifically regulated (Figure 6). *Reelin and PTEN* signalling are linked to neurogenesis, synaptic plasticity [26], neuronal migration and maturation [27] or local protein synthesis [28]. DHA signalling has previously been linked to LTP [29] and cell survival [30]. Thus these data predict an ongoing role of gene expression supporting synaptic growth and plasticity. Interestingly, PTEN associates with PSD95 and is recruited to the PSD in response to NMDA receptor activation [31] and has been linked to AMPAR trafficking [32]. These data interpreted alongside the observation that *Clathrin-mediated endocytosis* is a significantly regulated canonical pathway specifically within the 24 h-LRG set suggests that late phase LTP-related gene expression may contribute to homeostatic glutamate receptor regulation.

### 3. Ingenuity Pathway Analysis: Network analyses

To further explore our LRG datasets we used the network analysis tools of the IPA software to predict potential molecular networks functioning during the consolidation of LTP. These analyses revealed both effector pathways and highlight regulatory mechanisms engaged in response to LTP.

#### 3.1 Calcium signalling and LTP persistence

**Figure 7:**
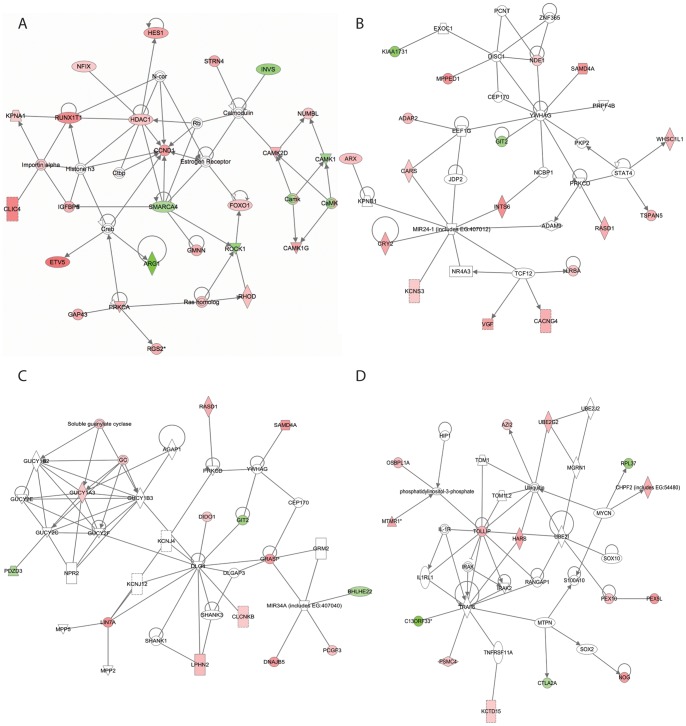
IPA network analysis at 5 h. The three highest scoring networks from LRG list are shown. Red, upregulated genes; green, downregulated genes. White open nodes, genes not present in LRG set but that interact with LRGs, as identified by the IPA Knowledge Base. A solid line denotes a direct functional interaction of the products of the two genes. A dotted line denotes an indirect interaction. An arrow indicates that a gene product “acts on” a target. *, Multiple affymetrix identifiers map to a single gene.

The most significant network formed at 5 h (5 h-Network 1; Figure 7A; Score  = 42; direct interactions) included the biological functions *Calcium, Protein kinase A and phospholipase C signalling*. This supports the primary conclusion from the IPA Canonical pathway and DAVID analysis that regulation of calcium signalling is a key marker of the 5 h-LRG set. Within this network calmodulin forms a central hub that is linked to a type 1 and type 2 calcium/calmodulin-dependent protein kinases cluster. Interestingly, this network also contains a hub centred on the transcription factor CREB, which interacts with the known LTP-related protein kinase PRKCA (aka PKCα) and its substrate Growth associated protein-43 (GAP43). Co-regulation of PRKCA and GAP43 is particularly pertinent as phosphorylation of GAP43 and the resulting enhanced release of glutamate have been mooted as a potential mechanism contributing to LTP persistence [33]. As CAMKI phosphorylates and activates synapsin I, a molecule also known to be LTP-regulated [34] and involved in neurotransmitter release, these data suggest a role for gene expression in supporting enhanced release of glutamate 5 h post-LTP, perhaps at downstream mossy fiber terminals in area CA3 [35].

CAMK signalling may contribute to LTP persistence in multiple ways. Recent literature has shown CAMKI-gamma (CAMKIG) to be enriched within the dentate gyrus and linked to dendritogenesis [36], [37] and CAMKI-alpha to axonal elongation [38]. As expression of CAMKIG was upregulated in our 5 h-LRG set as well as other molecules linked to postsynaptic reorganisation such as the protein kinase ROCKI (Rho-associated coiled-coil forming protein kinase 1) and its binding partner RHOD, this network may reflect LTP-responsive cytoskeletal reorganisation [39]. Together these data suggest that a key function of the 5 h-LRG network is related to synaptic reorganization.

#### 3.2 Negative regulation of gene expression at 5 h post-LTP

Several molecules involved in negative regulation of gene expression are present in 5 h-Network 1. For example, HistoneH3 forms a central hub and is linked to the epigenetic repressor of transcription HDAC1 (a member of the histone deacetylase complex) and its binding partner RUNX1T1, both of which are upregulated in our 5 h-LRG set. This complex removes acetyl moieties from histones, resulting in condensation of chromatin and transcriptional repression [40]. This network also includes transcriptional repressors such as NUMBL (numb homolog), which acts as a negative regulator of NFKB signalling [41] and HES1 (hairy and enhancer of split 1), which inhibits expression of genes regulated by bHLH (basic helix-loop-helix) proteins [42]. Furthermore, several specific transcriptional activators are *down*regulated in our 5 h-LRG set: for example, SMARCA4 (SWI/SNF related, matrix associated, actin dependent regulator of chromatin, subfamily a, member 4), a member of the CREST-BRG1 transcriptional coactivator complex [43], ROCK1, an activator of SRF [44] and CAMKI otherwise known as CREB kinase. Such molecular correlates of repression of gene expression are consistent with our observation that after the rapid LTP-induced increase in expression observed 20 min post-LTP (Figure 2; [13]) there is an attenuation of gene expression. The emerging theme of negative regulation of gene expression is further exemplified in a later highly significant network (5 h Network 4: Score 21; Figure 7D), where ubiquitin forms a central hub. This network predicts an LTP-associated increase in protein degradation which is mediated by upregulation of TOLLIP, (Tol interacting protein), a molecule which forms part of a complex which promotes endosomal trafficking of clathrin and ubiquitinated proteins to endosomes [45], and the ubiquitin-conjugating enzyme, UBE2G2 [46].

#### 3.3 Dynamic epigenetic regulation of LTP-related gene expression

In 5 h-Network 2 (Figure 7B; Score 33) the microRNA MIR24-1 formed a hub, while MIR34A is present within 5 h-Network 3 (Figure 7C; Score 18). While regulation of microRNA was not directly assessed in this study using Affymetrix RAT 230 2.0 arrays, the prediction generated by the IPA software that microRNA form central hubs within our networks is of particular note as microRNA are proposed to be central regulators of functionally related genes [47]. This is mediated by binding to the 3′untranslated region of target mRNAs, which results in translation repression and/or mRNA degradation. Interestingly, this process has been shown to occur in dendrites, and microRNA accordingly have also been proposed as key regulators of protein synthesis-dependent synaptic plasticity [48], [49], [50].

**Figure 8:**
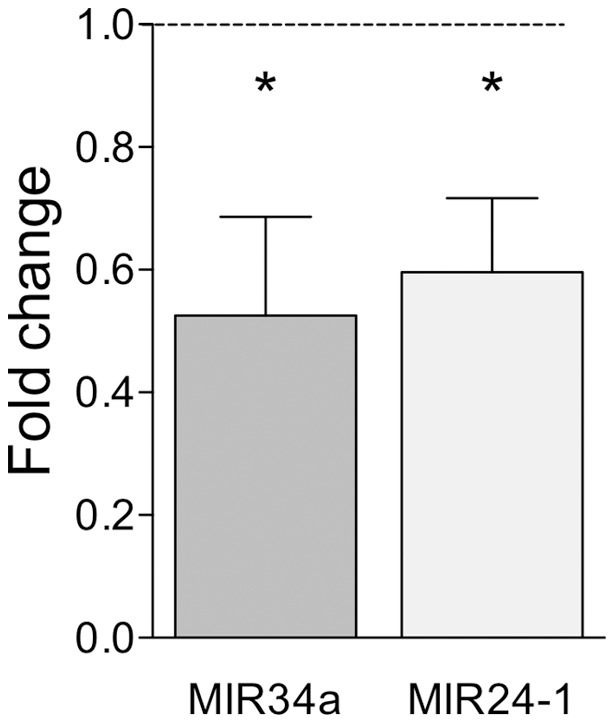
MicroRNAs identified by network analysis (5 h LRG-set) and validated by qPCR. Results are expressed as mean fold change +/− SEM. *p≤0.05, Student's t-test (n = 5–9). qPCR samples were normalised to the housekeeping gene Y1 using the 2^−ΔΔ^CT method.

As MIR24-1 is linked to 5 genes that are upregulated in response to LTP, the prediction from this network analysis is that MIR24-1 is downregulated in response to LTP, thus allowing the expression of a subset of linked genes. We tested this hypothesis using TaqMan microRNA qPCR and found a significant downregulation of MIR34A (0.53 ± 0.16 (average ± S.E.M), n = 5, p<0.05 two-tailed t-test) and MIR24-1 (0.59 ± 0.12, n = 9, p<0.05 two-tailed t-test) 5 h post-LTP (Figure 8). This is the first report of microRNA regulation following perforant path LTP in awake freely moving rats and is particularly relevant as the seed recognition sequence of the MIR34 family has recently been reported to rescue learning impairment [51]. Potentially, as antagonism of MIR24-1 has been shown to promote cell proliferation [52] and MIR34A regulates neural stem cell differentiation [53], these data suggest microRNA may also regulate LTP-related neurogenesis.

To investigate the potential mRNA targets of MIR34a and MIR24-1 we used the microRNA target prediction algorithms TargetScan, PicTar and miRanda. Prediction of MIR34a targets revealed 239 genes according to TargetScan, 438 according to miRanda, and 354 according to PicTar. Prediction of MIR24-1 targets revealed 329 genes according to TargetScan, 386 according to miRanda, and 369 according to PicTar.

Working on the principle that if a target is consistently predicted by multiple algorithms which use different selection criteria, it is more likely that the target prediction will be accurate, we investigated the intersection of the lists predicted by the three algorithms for each microRNA. This resulted in the identification of 30 genes that were predicted to be targeted by MIR34a and 19 genes predicted to be targeted by MIR24-1 (Table S7 and S8). Interestingly, a number of these genes are involved in synaptic transmission, specifically vesicle trafficking, endocytosis and exocytosis. However, when we screened our LTP datasets (5 h or 24 h) for the algorithm-predicted MIR34a and MIR24-1 target genes, we found no crossover between the groups. This does not discount the involvement of these microRNA in LTP consolidation but predicts that MIR34a and MIR24-1 at least contribute to LTP consolidation at the level of translational repression.

Taking an alternative approach, we used the web application miRvestigator Framework to test whether individual microRNA seed sequences were enriched within the mRNA of our LRG data sets. We found evidence for enrichment of the MIR330-3p seed sequence within our 5 h data set (p = 7.3e-04). Interestingly, the genes predicted to be targeted by MIR330-3p appear to primarily fall into the functional categories of transcription regulation and ubiquitination (Table S9).

**Figure 9:**
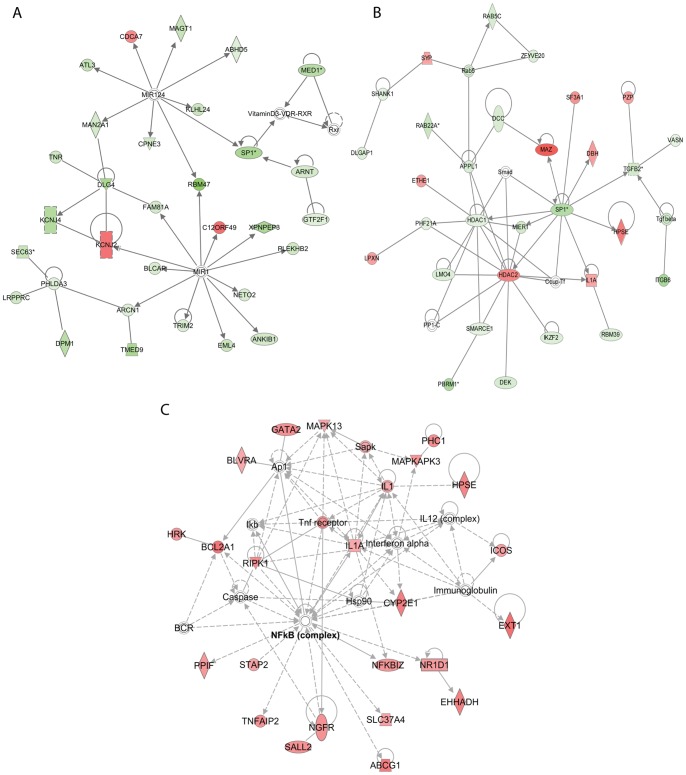
IPA network analysis at 24 h. The three highest scoring networks from the LRG list are shown. See Figure 5 legend for further details.

Epigenetic regulation of gene expression emerges as a central theme of the 24 h-LRG network analyses. Within 24 h-Network 1 (Figure 9A; all interactions; Score = 41), the microRNA MIR-1 and MIR-124 form central hubs, with the majority of the genes in this network downregulated. MIR-124 has been implicated in the long-term plasticity of synapses in the mature nervous system and as a central controller of neurogenesis. MIR-1, a microRNA which is expressed in human neuroblastoma cells [54], targets CREB1 and regulates cell growth [55], and differentiation of embryonic stem cells [56]. Interesting MIR-1 was also shown to inhibit apoptosis via modulating PTEN/Akt signalling, a canonical pathway we identified as LTP-regulated at 24 h post-LTP induction. Together these findings suggest a potential role for LTP-regulated microRNA in growth and/or neurogenesis.

HDACs also form central hubs within 24 h Network 1 (Figure 9B; direct interactions; Score 37). It is of note that HDAC2 is upregulated. This HDAC has previously been implicated in negative regulation of learning and memory [57]. In contrast HDAC1, which was upregulated within the 5 h-LRG set, is now downregulated, indicating a shift in the mode of action within the HDAC repressor complex similar to that shown to occur during development [58]. This network also contains evidence of direct transcriptional repression via downregulation of the transcription factor, SP1 (two probes: −1.5 fold), and downregulation of RBM39, which acts as an activator of the AP1-transcription factor complex. Taken together, these data suggest that regulation of microRNA, HDACs and transcription factors represents a homeostatic response 24 h post-LTP induction.

#### 3.4 Persistent involvement of NFKB in LTP-related gene expression

Having identified an LTP-related homeostatic response underpinned by gene expression, we reasoned that a targeted analysis of the subset of upregulated genes within the 24 h-LRG set may identify positive or effector functions. Using IPA Network analysis we found evidence for ongoing activity of the transcription factor NFKB (Figure 9C; Score 39; all interactions). This factor, previously implicated in the regulation of LTP-associated gene expression [59], forms the major hub of our most significant network generated from the 20 min LRG-dataset [13] and was identified in our Canonical Pathway analysis (Figure 4). Interestingly, the positive regulators of NFKB, AZ12 (5 h) and RIPK1 (24 h), were identified as upregulated post-LTP induction, while the negative regulator, NFKBIZ, is upregulated within the 24 h LRG-set suggesting multiple, dynamic mechanisms of NFKB activity regulation in the hours post-LTP induction. Interpretation of the genes present within the 24 h “upregulated only” network predicts that this gene network underpins two functions. One is modification of transcription mediated by, for example, recruitment of HDACs (NR1D1) and histone modification (IL1A and PHC1). The second is a theme of neuronal growth via both proliferation (STAP2) and differentiation mediated upregulation of NGFR (aka TNFR16; p75NTR) and its interacting protein SALL2. SALL2 is a transcription factor, which constitutively binds NGFR, and upon activation of the receptor dissociates and migrates to the nucleus and promotes cell cycle arrest and neurite outgrowth [60]. This analysis reinforces the conclusion that LTP is associated with ongoing regulation of gene expression and that the gene expression may function to promote neurotrophic processes.

## Discussion

We have used Ingenuity Pathway Analysis and DAVID to interpret how the differentially expressed genes identified at 20 min, 5 h and 24 h post-LTP induction at perforant path synapses *in vivo* are related. This analysis has confirmed that the persistence of LTP is associated with ongoing regulation of gene expression and has highlighted both an integrated network of regulatory mechanisms as well as effector mechanisms likely to contribute directly to the consolidation of LTP.

Our analysis has identified regulation of G-protein coupled signalling, calcium dynamics and ion channels as important biological functions that are regulated 5 h post-LTP induction, and clathrin-mediated endocytosis at 24 h. This suggests that the LTP-associated gene response may contribute to an alteration in the synaptic calcium response and potentially contribute to metaplasticity mechanisms proposed to facilitate or inhibit further plasticity within neuronal networks [7]. It is of note that the CaMK1 gene family, previously linked to synaptic reorganisation was differentially regulated. The conclusion that LTP-related gene expression contributes to structural alterations at synapses is strengthened by the observation that multiple pathways identified both at 5 h and 24 h converge on this or related functions. Furthermore, as a number of the differentially expressed genes (e.g. FOXO1, NUMBL, MIR24-1) or molecules predicted by the IPA software (e.g. MIR124) have reported roles in neurogenesis these highlight a potential role of gene expression in LTP-stimulated neurogenesis.

LTP persistence involves an intricate interplay of molecules and this is reflected in our results, which indicate that control of LTP-regulated gene expression occurs in multiple layers. We show evidence for ongoing positive regulation of gene expression via CREB (5 h-Network 1) and NFKB (24 h: Canonical Pathway; 24 h-Network “up only”), the functions of which appear to be at least in part the regulation of GAP43 and neurotrophic signalling respectively. These findings suggest that these two transcription factors that are activated immediately after LTP induction continue to play significant roles throughout the period of LTP consolidation.

Our network analysis has also highlighted mechanisms that account for the generalised downregulation of gene expression observed at 24 h. This response may contribute to resetting the transcriptional programme in response to LTP towards underlying homeostatic mechanisms, or it may facilitate expression of the “positive” LTP-related features, such as neurotrophin signalling, structural modification or neurogenesis. We identified multiple pathways that mediate downregulation of gene expression. These function by directly modulating protein levels either by ubiquitination, endocytosis, inhibition of protein synthesis or microRNA-driven translation arrest. Control of gene expression was also demonstrated through temporally controlled upregulation of histone acetylases and downregulation of transcription factors such as SP1. Importantly, as modification of histone acetylation has previously been shown to occur in other models of memory [57], [61], our data reinforce the importance of epigenetic mechanisms in synaptic plasticity. In summary, our data suggest that LTP-related gene expression contributes directly to both regulation of calcium dynamics as well as structural or neurogenic modifications of the network, which are likely to be involve ongoing activation of the CREB and NFKB transcription factors. Furthermore, we have identified a complex set of transcriptional regulatory mechanisms that appear to coalesce to form a transcriptional environment that facilitates the consolidation of LTP. These data should provide a solid platform for the development of biologically realistic neurogenetic computational models of memory storage processes.

## Materials and Methods

### Induction of LTP in freely moving animals

#### Ethics Statement

The surgical protocols, previously described in detail [13] were approved by the University of Otago Animal Ethics Committee (Permit number: 115–09) and were in accordance with New Zealand animal welfare legislation.

All experiments were conducted on perforant path dentate gyrus synapses in adult male Sprague-Dawley rats (400–550 g, 4–6 months old at the time of surgery). Teflon-coated stainless steel wire stimulating and recording electrodes were implanted bilaterally to establish perforant path-evoked field potentials, which were recorded in the dentate hilus. The methods of LTP induction are the same as those described in [3] and [13], with the exception that the animals were euthanized either 5 h or 24 h following LTP induction (n = 5 per group). In order to reduce variation all animals were tetanized unilaterally so that the unstimulated hemisphere could serve as a within-animal control. Our standard experimental protocols reliably produce an increase in the fEPSP that lasts for at least several weeks [3].

### RNA purification and microarray hybridization

RNA isolation and array hybridization were carried out as described previously [13]. RNA was isolated from individual snap-frozen dentate gyri using TRIzol reagent (Invitrogen) followed by purification through RNeasy Mini columns (Qiagen, USA). Samples meeting our quality control criteria (average RNA integrity number >8, [62]) were biotin-labeled and hybridized to Affymetrix RAT 230 2.0 arrays at the Centre for Proteomics and Genomics, University of Auckland. RNA isolation for microRNA analysis was performed similarly, but using Norgen Total RNA purification columns (Biotek Corporation) to retain these small RNA molecules.

### Microarray data analysis

All array data were assessed using the Bioconductor software packages [63] as described previously [13], with an initial evaluation performed using the *affyQCReport* package, which confirmed that all quality control measures were within the recommended ranges [64]. The *Robust Multichip Average (RMA)* package (version 1.16.0) was used to normalize the data derived from control and stimulated hemispheres 20 min [13], 5 h and 24 h datasets. As outlined before [13], inclusive LTP-related gene (LRG) sets suitable for network analysis (either direct or indirect interactions) were derived using dual selection criteria: a threshold fold change cutoff (1.15) and a moderated paired t-test between matched stimulated and control samples (significance criterion of p<0.05). The *t*-statistic was generated using the *Limma* package, which utilizes a standard error moderated across all genes using a simple Bayesian model and produces p-values with greater degrees of freedom and hence greater reliability [65]. More stringent selection criteria were not used in this study to avoid the risk of Type 2 error and unnecessarily limited datasets at 5 h and 24 h for network analysis. All data comply with MIAME guidelines and is available through *ArrayExpress* (Accession number to be confirmed).

### Real-time quantitative PCR: mRNA

Selected genes identified as differentially expressed by DNA microarray analysis were also analysed by quantitative PCR (qPCR). RNA was extracted as described above and reverse-transcribed to first strand cDNA using Superscript III (Invitrogen). Primers were designed using Primer 3 (http://frodo.wi.mit.edu/primer3/) and obtained from Sigma or Integrated DNA Technologies. Primer sequences are described in Table S6. qPCR was performed using SYBR green Roche mastermix on either an ABI 7300 or a Roche Lightcycler 480. qPCR samples were normalised to the housekeeping gene HPRT using the 2^-ΔΔ^CT method [66]. Standard curves were established and amplification and dissociation curves were analysed. Significance was assessed using Student's t-tests with the criterion set at p<0.05.

### Real-time quantitative PCR: microRNA

TaqMan® MicroRNA Assays were used to analyse MIR-34-a, MIR-24-1 and the endogenous control, Y1. RNA was extracted using TRIzol reagent (Invitrogen) followed by purification through Norgen Total RNA purification columns (Biotek Corporation) and reverse-transcribed using the TaqMan® MicroRNA Reverse Transcription Kit (Applied Biosystems) with an input of 10 ng total RNA per reaction. PCR reactions were carried out as per manufacturer's instructions. Ct values were normalized using the 2^-ΔΔ^CT method [66]. Significance was assessed using Student's t-tests with the significance criterion set at p<0.05.

### Identification of biologically relevant networks and biological pathways

*Ingenuity Pathway Analysis, version 7 (IPA; Ingenuity Systems, USA;* https://www.analysis.ingenuity.com *)* The individual LRG sets, containing Affymetrix identifiers and corresponding expression values (p<0.05) were uploaded to the IPA application and analysed, as described previously [13], to identify biological functions, canonical pathways and gene networks that may contribute to LTP maintenance and stabilization. IPA builds gene networks from input data utilizing the IPA knowledge base, which contains manually curated information sourced from published literature regarding the functions and interactions of molecules. A graphical representation of the predicted interactions of so-called focus genes is produced, which may contain molecules important to the structure of the network, but not identified as differentially expressed in the input data set (e.g. transcription factors activated by phosphorylation, or microRNA not assessed by mRNA array analysis). Each focus gene is represented as a node, where shape indicates functional groups (e.g. protein kinases) and colour indicates direction of regulation of the gene in the input dataset (green indicates downregulated; red indicates upregulated). Nodes are linked by lines (edges), which indicate proposed interactions. Solid lines indicate direct interactions while dotted lines indicate indirect interactions. In our analysis each network contained up to 35 genes and has an associated score derived from a p-value that indicates the expected likelihood of the focus genes being present in a network compared to that expected by chance. Scores of 3 or above have at least a 99.9% likelihood of not being generated by chance.

*The Database for Annotation, Visualization and Integrated Discovery* (DAVID; https://david.abcc.ncifcrf.gov/; [67]) was used for functional annotation clustering. This measures relationships among annotation terms on the basis of the degree of their co-association with genes within the inputted list. Enrichment scores of ≥1.3 are equivalent to non-log scale ≤0.05. A 2D-associated view tool was also used to examine the internal relationships among the clustered terms and genes.

### microRNA target prediction

The following computational algorithms were used to predict mRNA that may be targeted by microRNA of interest: TargetScan (www.targetscan.org/), PicTar (pictar.mdc-berlin.de/), and miRanda (http://www.ebi.ac.uk/). Lists of putative mRNA targets generated by each of the three algorithms were compared. To investigate microRNA seed sequence enrichment in our data we used the web application miRvestigator Framework (http://mirvestigator.systemsbiology.net/).

## Supporting Information

Figure S1 **Functional Annotation Clustering (DAVID Analysis) of LRGs at 5** **h.** Genes with annotations related to calcium and calmodulin. Enrichment score 1.42. Associations between the 5 h LRG sets and their annotations are illustrated above. The region in grey illustrates the common annotation across the cluster with areas in black illustrating the differences in annotation.
(TIF)

Figure S2 **Functional Annotation Clustering (DAVID Analysis) of LRGs at 5** **h.** Genes with annotations related to ion channels. Enrichment score 1.59. Associations between the 5 h LRG sets and their annotations are illustrated above. The region in grey illustrates the common annotation across the cluster with areas in black illustrating the differences in annotation.
(TIF)

Figure S3 **Functional Annotation Clustering (DAVID Analysis) of LRGs at 5** **h.** Genes with annotations related to the synapse. Enrichment score 1.37. Associations between the 5 h LRG sets and their annotations are illustrated above. The region in grey illustrates the common annotation across the cluster with areas in black illustrating the differences in annotation.
(TIF)

Figure S4 **Functional Annotation Clustering (DAVID Analysis) of LRGs at 24** **h.** Genes with annotations related to protein kinases, Enrichment score 1.98. Associations between the 24 h LRG sets and their annotations are illustrated above. The region in grey illustrates the common annotation across the cluster with areas in black illustrating the differences in annotation.
(TIF)

Figure S5 **Functional Annotation Clustering (DAVID Analysis) of LRGs at 24** **h.** Genes with annotations related to phosphorylation. Enrichment score 2.58. Associations between the 24 h LRG sets and their annotations are illustrated above. The region in grey illustrates the common annotation across the cluster with areas in black illustrating the differences in annotation.
(TIF)

Table S1 **Annotated genes differentially expressed at 5** **h post-tetanization ranked by p-value.**
(XLS)

Table S2 **Annotated genes differentially expressed at 24** **h post-tetanization ranked by p-value.**
(XLSX)

Table S3 **Expression of significant LRG genes across time.**
(DOC)

Table S4 **Annotated genes associated with 24** **h IPA Biological Function Protein Synthesis.**
(XLSX)

Table S5 **Annotated genes associated with 5** **h IPA Canonical Pathways.**
(XLSX)

Table S6 **List of primers used for real-time qPCR.**
(DOC)

Table S7 **Targets of miR-34a predicted by TargetScan, miRanda and PicTar.**
(XLSX)

Table S8 **Targets of miR-24-1 predicted by TargetScan, miRanda and PicTar.**
(XLSX)

Table S9 **Genes with sequence motifs from 5** **h LRG list corresponding to rno-MIR330-3p.**
(XLSX)
