# Supplementary figures and images for: Temporal Profiling of Gene Networks Associated with the Late Phase of Long-Term Potentiation In Vivo
Source: PLoS One. 2012 Jul 10;7(7):e40538. doi: 10.1371/journal.pone.0040538 (PMC3393663; doi:10.1371/journal.pone.0040538)

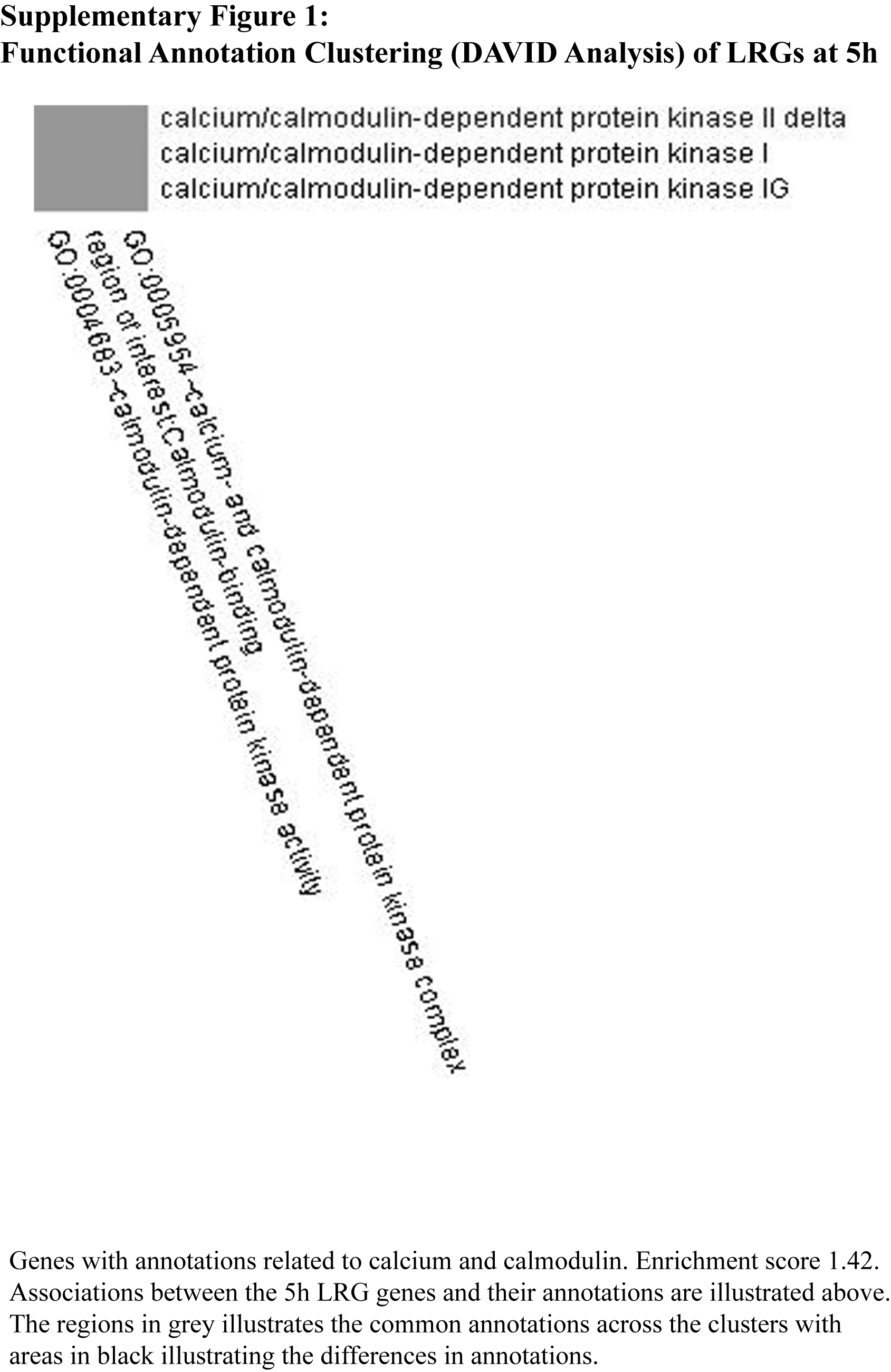

Supplement: Figure S1 — Functional Annotation Clustering (DAVID Analysis) of LRGs at 5 h. Genes with annotations related to calcium and calmodulin. Enrichment score 1.42. Associations between the 5 h LRG sets and their annotations are illustrated above. The region in grey illustrates the common annotation across the cluster with areas in black illustrating the differences in annotation. (TIF) [file pone.0040538.s001.tif]

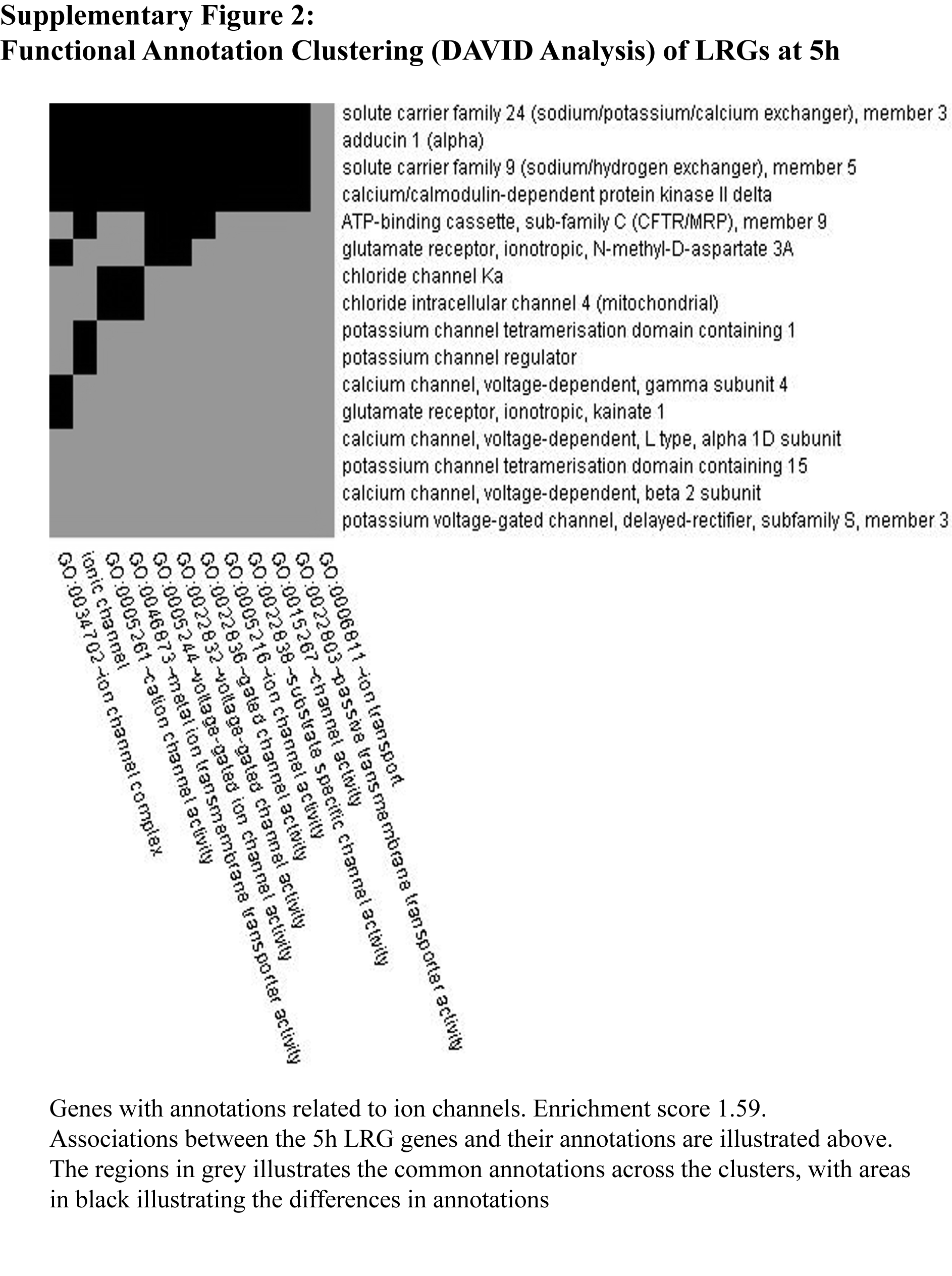

Supplement: Figure S2 — Functional Annotation Clustering (DAVID Analysis) of LRGs at 5 h. Genes with annotations related to ion channels. Enrichment score 1.59. Associations between the 5 h LRG sets and their annotations are illustrated above. The region in grey illustrates the common annotation across the cluster with areas in black illustrating the differences in annotation. (TIF) [file pone.0040538.s002.tif]

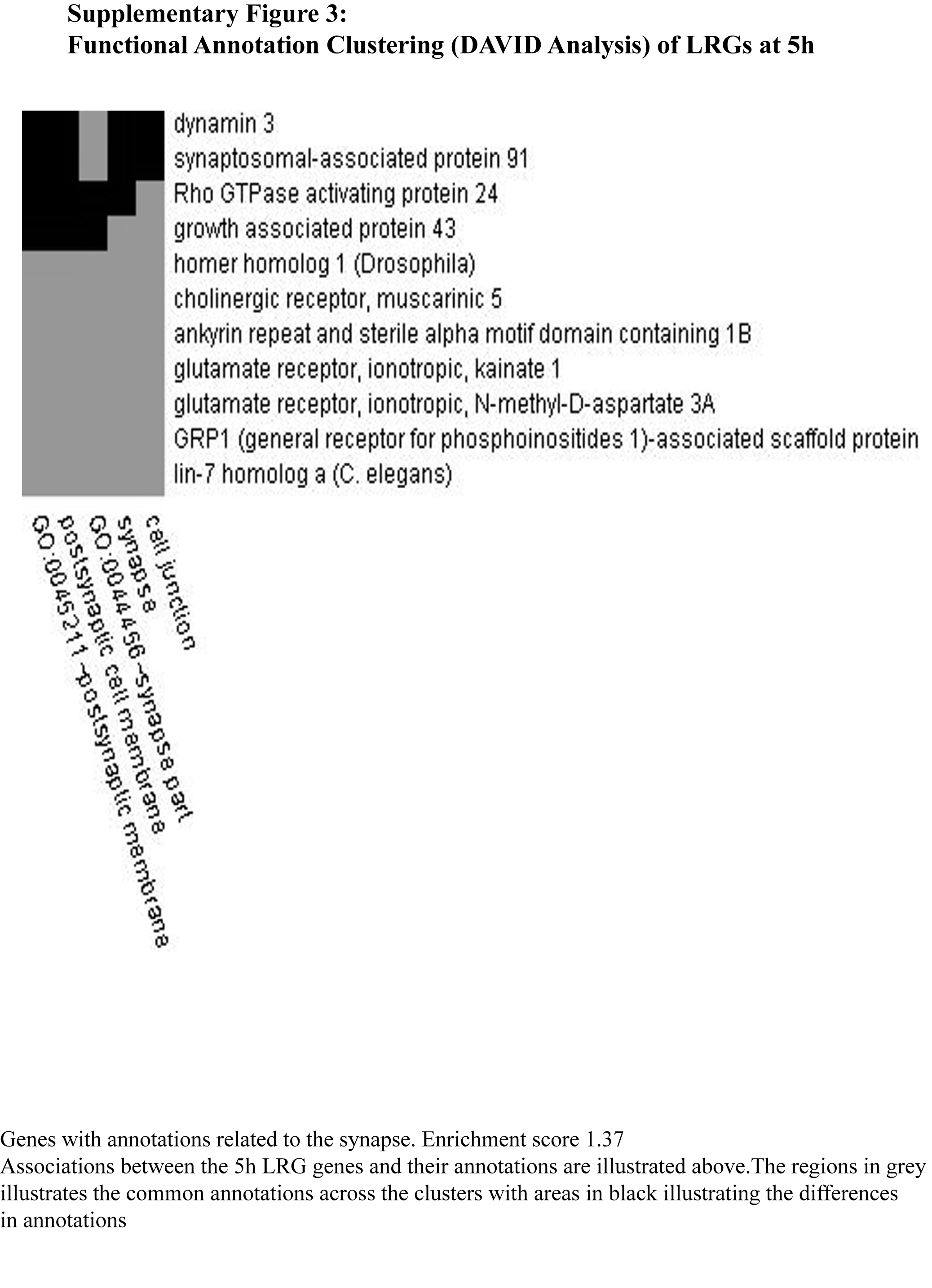

Supplement: Figure S3 — Functional Annotation Clustering (DAVID Analysis) of LRGs at 5 h. Genes with annotations related to the synapse. Enrichment score 1.37. Associations between the 5 h LRG sets and their annotations are illustrated above. The region in grey illustrates the common annotation across the cluster with areas in black illustrating the differences in annotation. (TIF) [file pone.0040538.s003.tif]

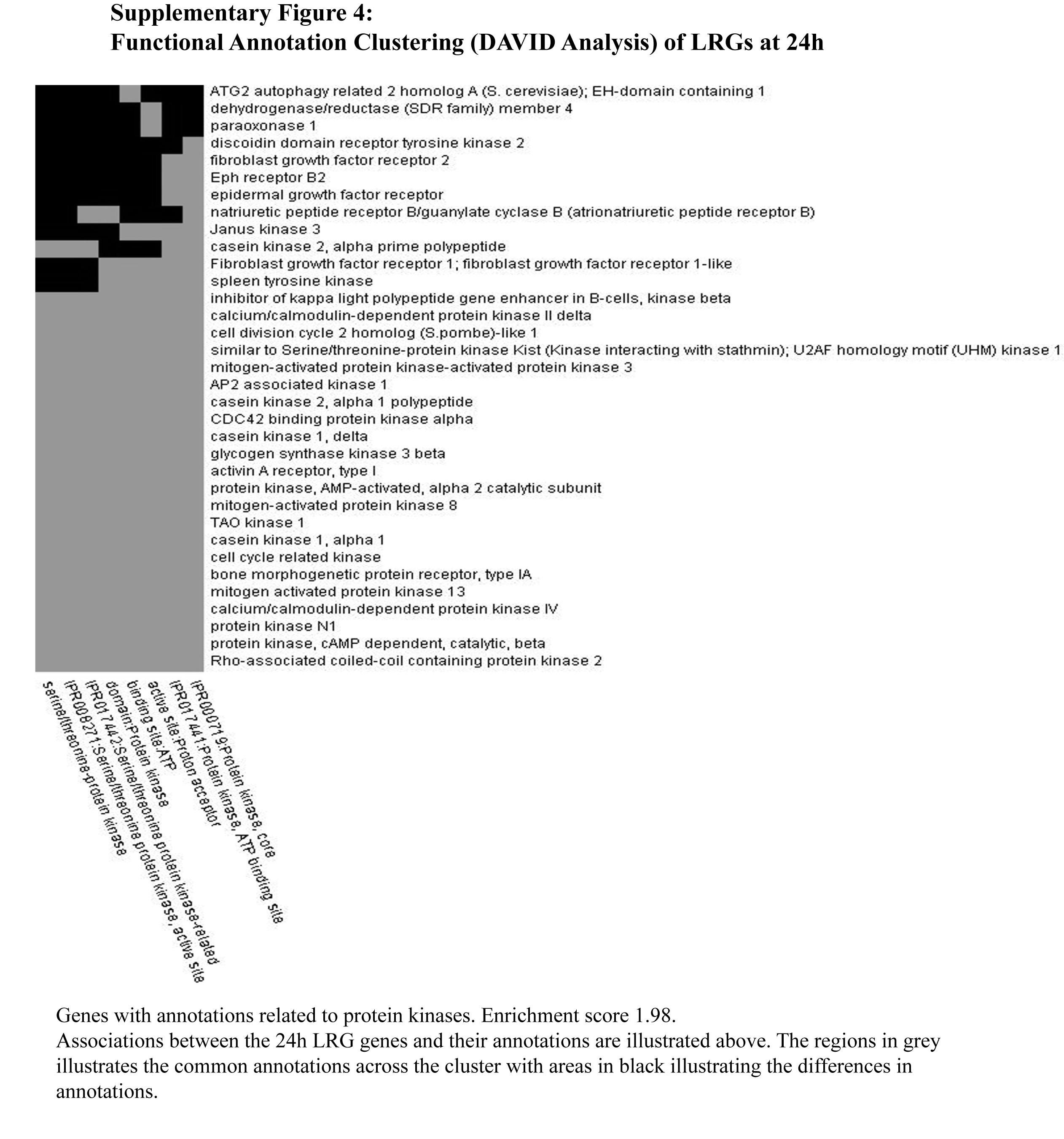

Supplement: Figure S4 — Functional Annotation Clustering (DAVID Analysis) of LRGs at 24 h. Genes with annotations related to protein kinases, Enrichment score 1.98. Associations between the 24 h LRG sets and their annotations are illustrated above. The region in grey illustrates the common annotation across the cluster with areas in black illustrating the differences in annotation. (TIF) [file pone.0040538.s004.tif]

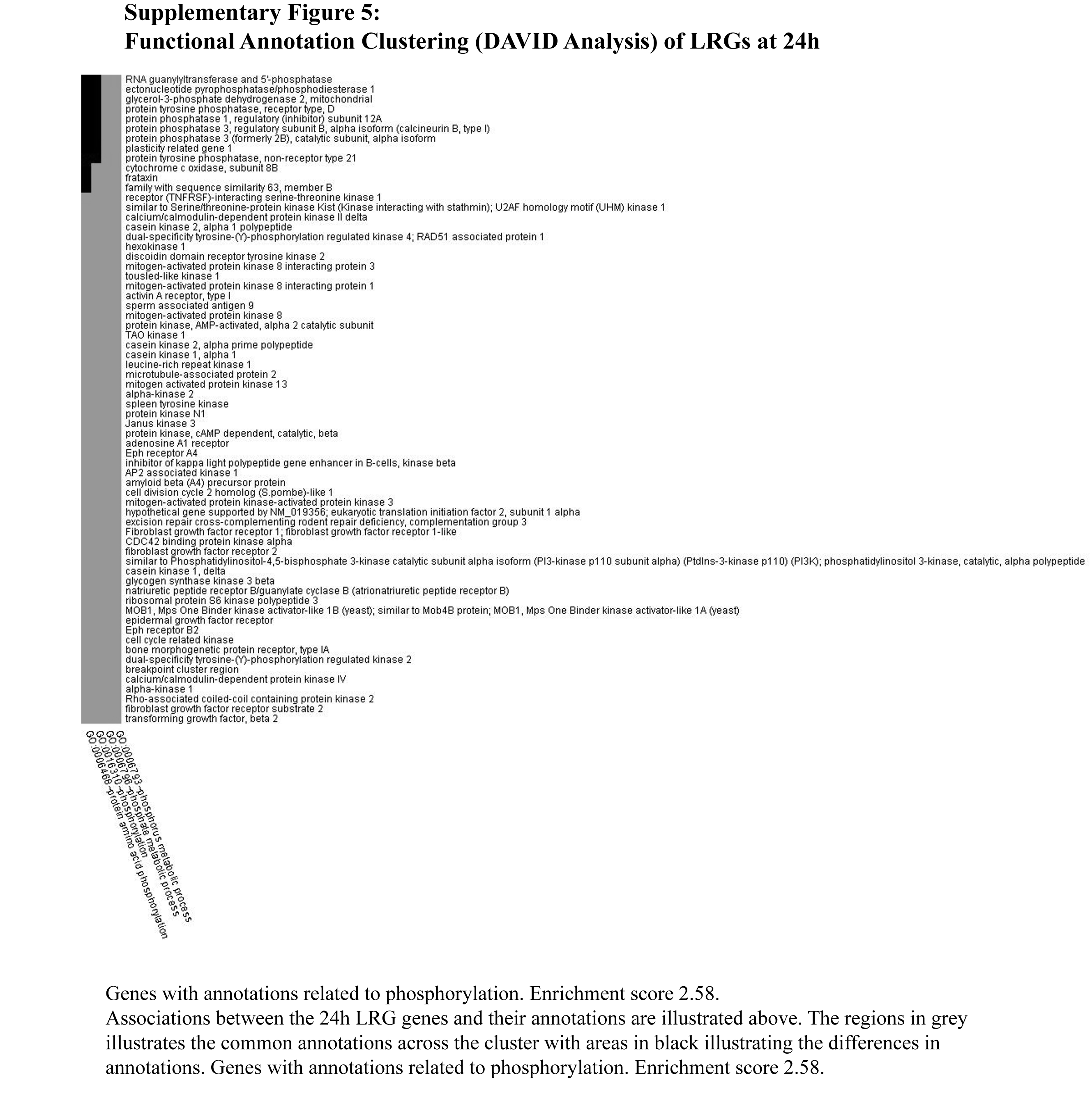

Supplement: Figure S5 — Functional Annotation Clustering (DAVID Analysis) of LRGs at 24 h. Genes with annotations related to phosphorylation. Enrichment score 2.58. Associations between the 24 h LRG sets and their annotations are illustrated above. The region in grey illustrates the common annotation across the cluster with areas in black illustrating the differences in annotation. (TIF) [file pone.0040538.s005.tif]
