# Supplementary material for: Temporal Profiling of Gene Networks Associated with the Late Phase of Long-Term Potentiation In Vivo
Source: PLoS One. 2012 Jul 10;7(7):e40538. doi: 10.1371/journal.pone.0040538 (PMC3393663; doi:10.1371/journal.pone.0040538)
Supplement: Table S3 — Expression of significant LRG genes across time. (DOC) [file pone.0040538.s008.doc]

Supplementary Table 3: Expression of significant LRG genes across time
