# Supplementary material for: Temporal Profiling of Gene Networks Associated with the Late Phase of Long-Term Potentiation In Vivo
Source: PLoS One. 2012 Jul 10;7(7):e40538. doi: 10.1371/journal.pone.0040538 (PMC3393663; doi:10.1371/journal.pone.0040538)
Supplement: Table S6 — List of primers used for real-time qPCR. (DOC) [file pone.0040538.s011.doc]

**Supplementary Table 6: List of primers used for real-time QPCR**

| **Gene Name** | **sense primer** | **anti-sense primer** |
| --- | --- | --- |
| HPRT | TGACACTGGTAAAACAATGCA | GGTCCTTTTCACCAGCAAGCT |
| TIMP1 | CGGTTCGCCTACACCCCAGC | CGTTCCTTAAACGGCCCGCGA |
| HOMER1 | GAAGTCGCAGGAGAAGAT | GAAGATCTCCTCCTGCTGATT |
| APP | GGTGCCTAGTTGGTGAGTTTGTAA | TCCTGGTGTAGAAACTTGCACTTG |
| LINGO1 | CCTGGTGCTGCTGTTTCT | CCCCATCCTCATATCATCTT |
| BHLHE22 | GCTGCTGAGGTTTTTGGAAG | GCACAGCACCCACTGAAAAA |
| DNAJB5 | AGCTCCTCACCGCAGCAC | AAACCCATTCAGCGTCCAC |
| HDAC1 | GTCCGGGCGGCGAGCAAGAT | TTCGGTGAGGCTTCATTGGGTGC |
| SP1 | ACCCCAAGCTGGTCGCAGGA | CCAGAGCCCCTCCCCTCACTG |
| KCNE2 | AGGTGAGCCTGGTGGGGTGA | CGTGCCCAGTCCCGTTGCTT |
